# Coagulopathy and Venous Thromboembolic Events Following Cytoreductive Surgery and Hyperthermic Intraperitoneal Chemotherapy

**DOI:** 10.1245/s10434-021-09941-9

**Published:** 2021-04-10

**Authors:** Paul Dranichnikov, Haile Mahteme, Peter H. Cashin, Wilhelm Graf

**Affiliations:** 1 Department of Surgical Sciences, Department of Colorectal Surgery, Uppsala University, Uppsala, Sweden; 2 Department of Surgical Sciences, Department of Colorectal Surgery, Uppsala University Hospital, Uppsala, Sweden; 3 Department of Surgery and Centre for Clinical Research, Västmanland Hospital Västerås, Västerås, Sweden

## Abstract

**Background:**

Coagulopathy after cytoreductive surgery (CRS) and hyperthermic intraperitoneal chemotherapy (HIPEC) is recognized but few details have been studied.

**Objectives:**

The aim of this study was to investigate changes in coagulation biomarkers and their predictive ability for venous thromboembolism (VTE).

**Methods:**

Patients undergoing CRS and HIPEC at Uppsala University Hospital, Sweden, from 2004 to 2014 were included in a prospective study of coagulation biomarkers. Prothrombin time international normalized ratio (PT-INR), activated partial thromboplastin time (APTT), fibrinogen, antithrombin, D-dimer, and platelets were sampled on postoperative days 1, 2, 5, and 10. Logistic regression analysis was used to evaluate predictive capacity for coagulation-related complications.

**Results:**

Overall, 380 patients were included (214 females, mean age 56 years); 38 patients had a history of thromboembolism and 57 were active smokers. Mean perioperative blood loss was 1228 mL and 231 (61%) received perioperative blood transfusions. PT-INR and APTT were elevated directly after surgery but returned to normal levels on postoperative day 5. Conversely, fibrinogen, platelet count, D-dimer, and antithrombin increased by postoperative day 5 and continued to increase up to day 10. There were 23 radiologically verified cases of VTE within 6 months. The multivariate analysis identified a completeness of cytoreduction score of 2–3 (*p* = 0.047) and day 2 D-dimer (*p* = 0.0082) as independent risk factors for postoperative VTE.

**Conclusion:**

Significant postoperative changes in coagulation biomarkers occur with dynamic changes over 10 days postoperatively. The incidence of symptomatic VTE was low. Residual tumor at completion of surgery and elevated D-dimer on day 2 were independent risk factors for postoperative VTE.

Postoperative coagulopathy and venous thromboembolism (VTE) are well-known manifestations after major cancer surgery and the risk for thromboembolic complications is higher for cancer patients compared with non-cancer patients undergoing similar surgery. This risk is estimated as twofold for deep vein thrombosis (DVT) and threefold for pulmonary embolism (PE).^1^^,^^2^ VTE is considered one of the main causes of postoperative mortality in cancer patients,^3^ and there is a significantly higher mortality risk if the patient has a concurrent VTE complication.^4^

The incidence of DVT is about 26% in abdominal surgery and 14% in gynecological surgery without thromboprophylaxis. This risk is considered to be even higher in cancer patients.^5^ With thromboprophylaxis, the risk of thromboembolic complications differs extensively depending on the type of malignancy and disease duration.^6^ Some tumors, such as gastrointestinal carcinomas and carcinomas of the ovary and lung, are commonly associated with various thromboembolic complications. An autopsy study on patients who died of pancreatic cancer found a 30% incidence of thrombosis.^7^ However, not all VTEs are symptomatic. and according to Kodama et al., the risk of symptomatic VTE is highest 2 weeks postoperatively.^8^

Peritoneal surface malignancy (PSM) is considered a locoregional manifestation of cancer metastases from various malignancies, including colorectal, ovarian, appendiceal, and mesothelioma, which, prior to the development of cytoreductive surgery (CRS) and hyperthermic intraperitoneal chemotherapy (HIPEC), was treated with palliative systemic chemotherapy or best supportive care.^9^–^11^ Although the treatment of PSM with CRS and HIPEC has improved survival, the procedures are associated with high morbidity levels and a variety of physiological changes, as well as prolonged hospital stays, affecting the cardiovascular system and coagulation cascade.^12^–^14^ The risk for postoperative VTE in PSM patients has been estimated as high as 30–50% without prophylaxis;^15^ however, with prophylaxis, Khan et al. reported 5.6% VTEs within 60 days postoperatively in a cohort of 447 patients. Moreover, the same study concluded that compliance with current guidelines for extended postoperative prophylaxis was associated with reduced VTEs.^11^

The occurrence of thromboembolic complications after CRS and HIPEC or any other major surgery depends strongly on the balance between tissue damage, coagulation, and the thrombolytic–fibrinolytic system function.^16^ To date, no studies have investigated postoperative predictive coagulation biomarkers for VTE following CRS and HIPEC. However, D-dimer is a well-known stable end product of fibrin degradation and an increase in its levels has been widely used for the screening of VTE.^8^ Kodama et al. argued that a high plasma D-dimer on postoperative day 3 was one of the independent risk factors for postoperative VTE following gynecological cancer surgery.^8^

The aim of this study was to investigate changes in coagulation biomarkers after CRS and HIPEC and their predictive abilities for VTE.

## Patients and Methods

### The Cohort

All patients with a Swedish social security number who underwent open, elective CRS and HIPEC at Uppsala University Hospital (UAS) between 2004 and 2014 were included in the study cohort. Demographics, laboratory values, and surgical factors were retrieved from the local HIPEC registry. Data for morbidity-related readmission within 6 months after CRS and HIPEC were retrieved from the Swedish In-Patient Register, as well as the Cause of Death Register, by using the International Classification of Diseases (ICD) operation code JAQ10 (intraoperative hyperthermic chemotherapy in the abdominal cavity). All hospitalizations for VTE in Sweden within 6 months after CRS and HIPEC, regardless of where the patient was treated, were thus recorded using the Swedish In-Patient Register.

Baseline characteristics, including body mass index (BMI), smoking habits, medication that might affect hemostasis, and previous thromboembolic events, previous abdominal surgery prior to CRS and HIPEC, and previous chemotherapy for PSM, were recorded for all patients. During surgery, the lithotomy position was not routine but was used selectively when a colorectal anastomosis was anticipated.

Postoperative adverse events were classified according to the Clavien–Dindo classification,^17^ but only grades III–V were included in the analysis. The Caprini score (2005)^18^ was used to assess the postoperative risk for VTE. A retrospective application of the Caprini score was conducted. If no information regarding a risk factor was found, the risk factor was scored as 0.

The time frame for postoperative adverse events was expanded to 6 months in order to investigate possible late HIPEC-related readmissions, as an earlier study found that the readmission rate is quite high due to late adverse events.^19^

This study was approved by the Regional Ethics Board, Uppsala, Sweden (reference no. 2007/073).

### Coagulation Monitoring

Coagulation tests were categorized in three groups: (1) the integrity of the extrinsic and common pathways by following the dynamics of the prothrombin time international normalized ratio (PT-INR), and the activity of the intrinsic and common pathways by following the dynamics of activated partial thromboplastin time (APTT); (2) the thrombogenic activity (fibrinogen, erythrocyte count, platelet count, and D-dimer); and (3) antithrombogenic activity (antithrombin). Routine preoperative blood tests (hemoglobin, erythrocyte count, platelet count, leukocyte count, and PT-INR) were performed on all patients upon admission. The following blood tests were sampled on postoperative days 1, 2, 5, and 10: PT-INR, APTT, fibrinogen, antithrombin, D-dimer, and platelet count. In addition, hemoglobin, erythrocyte, platelet, and leukocyte counts were retrieved preoperatively and at discharge from hospital.

Perioperative coagulation status was evaluated using a thromboelastogram (TEG) in order to calculate and manage the need for transfusion. In general, 300 mL erythrocyte concentrate per 1000 mL perioperative bleeding, and 300 mL plasma per 500 mL perioperative bleeding, was administered.

### Perioperative Variables

The following parameters were registered: primary tumor origin, liver resection, splenectomy, perioperative blood loss, perioperative erythrocytes and plasma transfusion, HIPEC regimen, intra-abdominal temperature during HIPEC, early postoperative intraperitoneal chemotherapy (EPIC) if administered, duration of surgery, duration of in-hospital care, adverse events, interventions, duration of thromboprophylaxis, postoperative thromboembolic events, adjuvant chemotherapy, and mortality.

Thromboprophylaxis treatment was administered according to the routine performed at Uppsala County Hospital, as a subcutaneous injection of Klexane (Sanofi Paris, Paris, France) 100 mg/mL, 0.4 mL once daily for a total treatment period of 4 weeks. All patients started thromboprophylaxis treatment the day before surgery and continued daily for a planned period of 4 weeks postoperatively. In addition, mechanical prophylaxis using sequential compression devices were used as part of the postoperative care, in addition to compression garments and routine physiotherapy.

### Statistics

Statistical analysis was performed using Statistica 64 software for Windows (version 13.3’ Dell Software, Round Rock, TX, USA). Descriptive statistics are presented as mean, median, percentage, and range. Risk analysis was performed using univariate logistic regression on each possible predictor for postoperative thromboembolic events. Thereafter, a multivariate logistic regression was performed including all univariate factors with a *p* value < 0.05. Adjustments were made using multiple imputations on PT-INR, APTT, fibrinogen, antithrombin, and D-dimer due to missing data (last value carried forward + mean + median/3). Odds ratios (OR) and corresponding 95% confidence intervals (CI) were calculated and statistical significance was defined at as *p* < 0.05.

## Results

### Demographics

A total of 380 patients with PSM were included (Table 1). Patients were 56% females (*n *= 214) and 44% males (*n *= 166). Mean Karnofsky performance status was 96; the majority (73%) patients had a score of 100 (*n *= 279). Fifty-two percent of the cohort had appendiceal cancer as the primary tumor site (*n *= 197), 32.6% had primary tumor from colorectal origin (*n *= 124), 6% from gynecological origin (*n *= 23), 5% mesothelioma (*n *= 19), 3% from the small intestine (*n *= 11), and only 1.5% had gastric cancer (*n *= 6). Thirty-one percent of the cohort had received preoperative chemotherapy within 3 months prior to HIPEC (*n *= 116). The majority of preoperative chemotherapy recipients had primary tumor from colorectal origin (51%, *n *= 59). Groups with other primary tumors had less neoadjuvant recipients, with 27% in the appendix group (*n *= 31), 10% in the gynecological group (*n *= 12), 5% in the gastric group (*n *= 6), 4% in the mesothelioma group (*n *= 5), and 3% in the small intestine group (*n *= 3).

**Table 1:** General demographics and surgical variables

Variables	Results
Age, years [mean (range)]	56 (22–77)
BMI [mean (range)]	26 (17–40)
Female/male	214 (56)/166 (44)
Smoking habits	
Previous smokers	55 (14)
Active smokers	34 (9)
Never smoked tobacco	291 (77)
Cardiovascular comorbidity	109 (29)
Thromboembolism predisposition	40 (11)
Primary tumor site	
Appendix	197 (52)
Colorectal	124 (32.6)
Gynecological	23 (6)
Mesothelioma	19 (5)
Small intestine	11 (3)
Gastric	6 (1.5)
Preoperative chemotherapy for PM disease (within 3 months prior to surgery)	
Yes	116 (31)
No	264 (69)
ASA score	
1–2	347 (91)
3	34 (9)
Liver resection	89 (23)
Splenectomy	149 (39)
PCI	
1–20	216 (57)
21–39	153 (40)
Unidentified	11 (3)
CCS	
0–1	335 (88)
2–3	45 (12)
Operation duration, hours [mean (range)]	9.3 (4–18)
Estimated blood loss, mL [mean (range)]	1228 (25–15,325)
< 2000	298 (78)
≥ 2000	82 (22)
HIPEC regimen	
Oxaliplatin	186 (49)
Mitomycin C	114 (30)
Cisplatin/doxorubicin	80 (21)
CRS/HIPEC + EPIC	101 (27)
Caprini Score [median (range)]	8 (6–15)

Data are expressed as *n* (%) unless otherwise specified

*BMI* body mass index, *PM* peritoneal metastasis, *ASA* American Society of Anesthesiologists, *PCI* Peritoneal Cancer Index, *CCS* completeness of cytoreduction score, *HIPEC* hyperthermic intraperitoneal chemotherapy, *CRS* cytoreductive surgery, *EPIC* early postoperative intraperitoneal chemotherapy

In the entire cohort, a complete cytoreduction to microscopic disease burden (CC0) was achieved in 264 patients (69%). Mean duration of surgery was 9.3 h (range 4–18 h), and mean length of hospital stay was 25.8 days (range 10–124 days). In addition, 27% of the cohort received adjuvant chemotherapy (*n *= 102).

### Incidence of Venous Thromboembolism

In total, 6% of VTEs occurred within 6 months (*n *= 23) (Table 2). These patients had a median Caprini score of 8 (range 7–13), compared with 94% of non-VTE patients who had a median Caprini score of 8 (range 6–15). VTEs occurred in 5.6% (11/197) of patients with appendix tumors. VTE distribution among other primary tumor groups was 6.5% in the colorectal group (8/124), 33% in the gastric group (2/6), 4% in the gynecological group (1/23), and 5% presented in the mesothelioma group (1/19), while no VTEs presented in the small intestine group. Twelve of the VTEs occurred in hospital and 11 occurred after discharge. None of the VTEs were diagnosed in the upper extremities.

**Table 2:** Postoperative outcome and survival

Variables	Results
Postoperative care at the ICU, days	348 (92)
0	32 (8)
0.5–1	296 (78)
2	34 (9)
≥ 3	18 (5)
In-hospital reoperation	35 (9)
Morbidity requiring interventions (Clavien–Dindo III–V)	80 (21)
Number of days hospitalized post HIPEC [mean (range)]	26 (10–124)
Postoperative transfusion	
Packed erythrocytes, mL; *n *= 222 (58%) [mean (range)]	796 (300–4800)
0 packs	158 (41.5)
1–5 packs	206 (54)
> 5 packs	16 (5)
Anticoagulant therapy	
4 weeks postoperatively (routine)	308 (81)
Prolonged duration (> 5 weeks)	55 (14.5)
Short duration (2–3 weeks)	17 (4.5)
VTE within 6 months post HIPEC	23 (6)
Pulmonary embolism	12 (3)
Deep vein thrombosis	11 (3)
Mean time to VTE, days (range)	64 (1–113)
Alive at study cut-off date (30 April 2018)	182 (48)
5-year survival	219 (58)
In-hospital mortality	4 (1)
Dead within 6 months	16 (4)

Data are expressed as *n* (%) unless otherwise specified

*ICU* intensive care unit, *HIPEC* hyperthermic intraperitoneal chemotherapy, *VTE* venous thromboembolism

In addition, seven cases of arterial thrombotic events (ATE) were diagnosed. Five of these events occurred in hospital, of which three were myocardial infarction (MI), one was a cerebrovascular insult (CVI), and one was a thrombosis in the left external iliac artery. Two ATEs occurred within 6 months of the first discharge—both were CVIs.

Of the 4% of patients with inflammatory bowel disease (IBD; *n *= 16), none developed a VTE.

A total of 26 patients had > 3000 mL intraoperative blood loss, of whom four patients had a temporary interruption of postoperative anticoagulant therapy (range 1–3 days); however, none of these four patients developed VTE within 6 months.

Only 4% of patients received a shortened postoperative anticoagulant therapy (2–3 weeks) due to the risk of hemorrhage (*n *= 17); however, none of those patients developed VTE within 6 months of their CRS and HIPEC.

### Dynamics and Univariate Predictive Analysis of Postoperative Coagulation Biomarkers

**Fig. 1:**
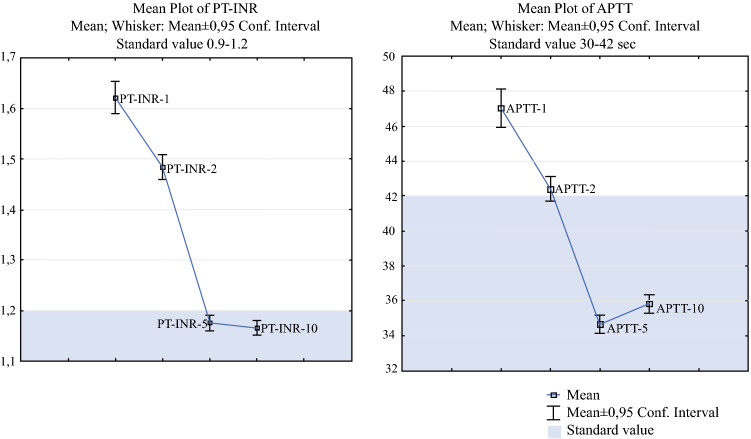
The dynamics of pro-bleeding coagulation markers in relation to time and standard value. *PT-INR* prothrombin time international normalized ratio, *APTT* activated partial thromboplastin time, *Conf. Interval* confidence interval

**Fig. 2:**
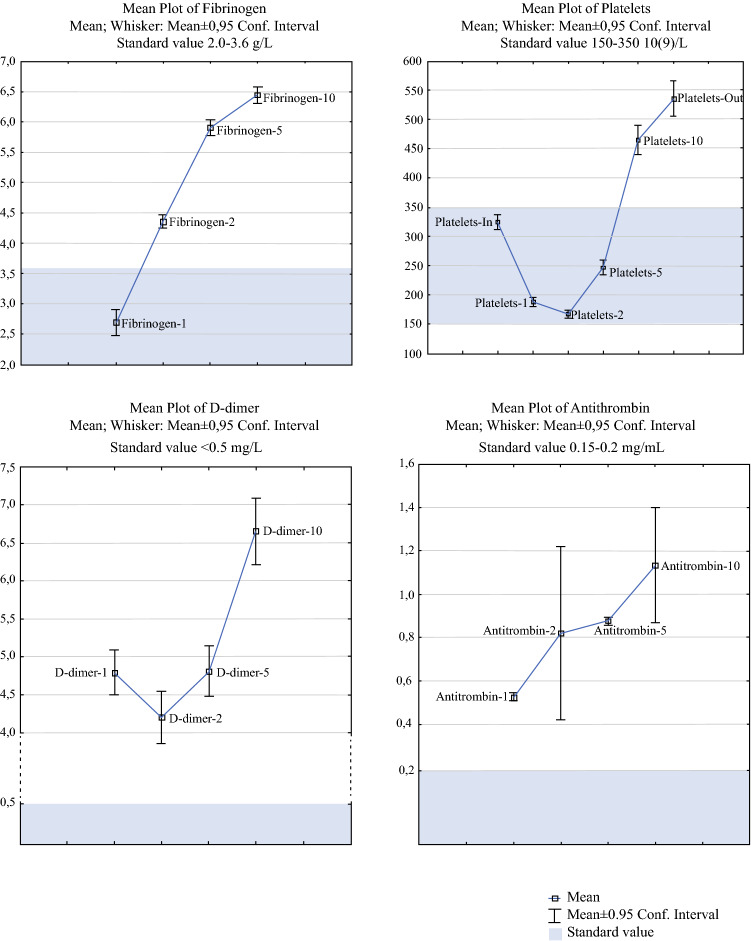
The dynamics of prothrombotic coagulation markers in relation to time and standard value. *Conf. Interval* confidence interval

PT-INR and APTT were elevated directly after surgery but returned to normal levels on postoperative day 5 (Fig. 1). Conversely, fibrinogen, platelet count, D-dimer, and antithrombin showed an increased level on postoperative day 5 and a further increment up to day 10 (Fig. 2). Univariate analysis on day 1 showed that an elevated level of D-dimer was a significant risk factor for VTE (OR 0.88, 95% CI 0.79–0.98, *p *= 0.02) but this was excluded from further analysis due to a large amount of missing data (36%, *n *= 135). The remaining markers sampled on day 1 did not indicate any significant risk for VTE (PT-INR, *p *= 0.88; APTT, *p *= 0.53; fibrinogen, *p *= 0.51; antithrombin, *p *= 0.96; platelet count, *p *= 0.39). Elevated D-dimer on day 2 indicated an increased risk for VTE (OR 1.12, 95% CI 1.04–1.22, *p *= 0.004).

The remaining biomarkers on day 2 were not associated with an increased risk for VTE. (PT-INR, *p *= 0.84; APTT, *p *= 0.81; fibrinogen, *p *= 0.89; antithrombin, *p *= 0.71; platelet count, *p *= 0.92). Likewise, there was no correlation between the same coagulation biomarker panel on days 5 and 10 and an increased risk for VTE. There was no correlation between blood tests at discharge (hemoglobin, *p *= 0.78; erythrocyte count, *p *= 0.37; platelet count, *p *= 0.86; leukocyte count, *p *= 0.55) and an increased risk for VTE.

### Risk Analysis

Erythrocyte count upon admission was a predictor for VTE within 6 months after CRS and HIPEC in univariate logistic regression analysis (OR 0.36, 95% Cl 0.14–0.94, *p *= 0.03) (Table 3). Other blood tests upon admission had no correlation to the risk for VTE.

**Table 3:** Logistic regression of venous thromboembolism events within 6 months of CRS and HIPEC

	Univariate analysis [OR (95% CI)]	*p* value	Multivariate analysis [OR (95% CI)]	*p *value
Age at treatment	0.96 (0.93–1.00)	0.055		
Sex: male versus female	0.57 (0.24–1.35)	0.20		
BMI, kg/m^2^	0.96 (0.86–1.07)	0.50		
Active smoker	0.96 (0.21–4.31)	0.96		
Previous smoker	1.46 (0.58–3.68)	0.41		
Non-smoker	Reference			
Cardiovascular comorbidity	1.35 (0.55–3.28)	0.50		
Caprini score	1.26 (0.98–1.61)	0.06		
Primary tumor site^a^				
Colorectal	*Reference*			
Appendix	1.16 (0.45–2.98)	0.92		
Gynecological	1.51 (0.18–12.74)	0.78		
Mesothelioma	1.24 (0.14–10.52)	0.98		
Preoperative intravenous chemotherapy, within 3 months	1.22 (0.50–2.98)	0.64		
HIPEC: oxaliplatin	*Reference*			
HIPEC: cisplatin + doxorubicin	1.31 (0.40–4.19)	0.66		
HIPEC: mitomycin C	1.05 (0.40–2.76)	0.86		
PCI	1.00 (0.96–1.04)	0.98		
CCS				
0–1	*Reference*		*Reference*	
2–3	2.87 (1.07–7.73)	0.03	2.78 (1.01–7.62)	0.047
Splenectomy	2.11 (0.90–4.95)	0.08		
Liver resection	1.81 (0.74–4.43)	0.18		
HIPEC + EPIC	1.51 (0.62–3.68)	0.36		
Operation duration	0.88 (0.76–1.03)	0.12		
Estimated blood loss, mL	0.99 (0.99–1.00)	0.06		
ASA score: 3 versus 1–2	1.57 (0.44–5.60)	0.48		
Postoperative ICU: ≥ 2 days versus 0–1 days	0.59 (0.13–2.63)	0.49		
Erythrocyte count upon admission^b^	0.36 (0.14–0.94)	0.03	0.39 (0.15–1.05)	0.061
D-dimer postoperative day 2	1.12 (1.04–1.22)	0.004	1.12 (1.03–1.21)	0.0082
Clavien–Dindo grade III–IV	1.98 (0.85–4.60)	0.11		
Adjuvant chemotherapy	0.74 (0.26–2.06)	0.57		

*CRS* cytoreductive surgery, *HIPEC* hyperthermic intraperitoneal chemotherapy, *OR* odds ratio, *CI* confidence interval, *BMI* body mass index, *PCI* Peritoneal Cancer Index, *CCS* completeness of cytoreduction, *EPIC* early postoperative intraperitoneal chemotherapy, *ASA* American Society of Anesthesiologists, *ICU* intensive care unit, *VTE* venous thromboembolism

^a^Small intestine group was excluded due to the absence of VTEs. The gastric cancer group was excluded due to the small number of patients (*n *= 6)

^b^Erythrocyte count cut-off value: 4.30 × 10^12^/L

Incomplete cytoreduction (CC2–3) was a significant risk factor for VTE (OR 2.87, 95% CI 1.07–7.73, *p *= 0.03), whereas no other surgical factors influenced the risk for VTE (Table 3).

The multivariate logistic regression analysis found incomplete cytoreduction and D-dimer on day 2 to be independent risk factors for VTE within 6 months. D-dimer levels on day 1 were significant in the univariate analysis, but due to missing data and covariation with D-dimer on day 2, these were excluded from the multivariate analysis.

## Discussion

The general morbidity after CRS and HIPEC has been the subject of many studies; however, little is known about hemostasis imbalance and coagulopathy. Two important risk factors for postoperative VTE were identified in this study—incomplete cytoreduction and elevated D-dimer on postoperative day 2.

In this prospective study on coagulation biomarkers, thromboembolism after CRS and HIPEC was assessed by repeated blood tests and retrieval of the VTE endpoint at the national level. Analyses showed that PT-INR and APTT were elevated directly after surgery but returned to normal levels by postoperative day 5 (Fig. 1), suggesting an increased bleeding diathesis during the first few postoperative days. Conversely, fibrinogen, platelet count, D-dimer, and antithrombin increased by day 5 and continued to increase up to the last day of the study, predicting a possible onset for an elevated risk for thromboembolic complications (Fig. 2). Interestingly, one interventional randomized trial has aimed to mitigate intraoperative-acquired fibrinogen deficiency in order to prevent bleeding.^20^

The cumulative incidence of VTE within 6 months after CRS and HIPEC was 6%. Considering all pre-, peri- and postoperative risk factors, this rate is in the middle range compared with previous studies (3–14%).^21^–^24^ Khan et al. argued that VTEs are relatively common after CRS and HIPEC and thus reported a 5.6% VTE rate after CRS and HIPEC in a cohort of 447 patients, within 60 days postoperatively.^11^ Although the main mechanisms involved in perioperative hypercoagulation stimuli are still unclear,^7^ many studies suggest a multifactorial influence on the coagulation system.^25^–^27^

Using the Caprini score to take into account many of the known risk factors for VTE, there was a non-significant predictive result; however, this was close, with a *p* value of 0.06. In a larger cohort, this may prove to be significant; however, regardless, in this cohort, it proved less useful than expected considering the many factors included. One caveat is that this risk score was retrospectively applied, which is known to be a suboptimal scoring application as some of the variables are not systematically examined (e.g. history of varicose veins).

Connolly et al. and Falanga et al. categorized thrombotic risk factors in cancer patients in general into three groups: patient-related (high age, bed rest, obesity, previous thrombosis, heredity for thrombosis, high leukocyte and platelet count, and comorbidity); cancer-related (site and stage of cancer, inflammatory component, necrotic tumor); and, finally, treatment-related (surgical trauma, hospitalization, and prolonged immobilization, chemotherapy, etc.)^6^^,^^28^ The extent and duration of surgery, as well as vascular injuries and degree of tissue damage, reduced liver perfusion, excessive blood loss, massive fluid replacement during CRS and HIPEC, and postoperative immobilization, are some of the perioperative thrombogenic factors that might shift hemostasis out of balance and increase the risk for postoperative thromboembolic complications. Furthermore, the risks are considered to be higher for patients with predisposing conditions, as well as comorbidity and lifestyle, including high BMI and the use of tobacco.^29^^,^^30^

Using the classification of thrombotic risk factors in cancer patients by Falanga et al.,^28^ our results failed to show that patient-related factors were related to a significant risk for VTE within 6 months of CRS and HIPEC (Table 3). Although it is well known that the risk of VTE is increased approximately threefold for patients with IBD,^31^ none of the 4% of IBD patients in this study (*n *= 16) developed VTE within 6 months postoperatively.

While platelets are the main cellular component in the coagulation cascade, by interacting with coagulations factors and resulting in a thrombus in the blood vessel at the site of injury,^32^ our study results showed no significantly increased risk for VTE when the platelet count was elevated. Figure 2 demonstrates the dynamics of platelets and other coagulation markers in relation to time. On the other hand, Litvinov et al. suggested that erythrocytes or red blood cells (RBCs) play a rheological role in coagulation by involving laminar shearing with platelet marginalization, as well as interacting with endothelial cells and platelets, which may be involved in thrombosis.^33^ Our univariate analysis on erythrocyte count upon admission prior to CRS and HIPEC, using VTE risk as the endpoint, showed a significant risk (OR 0.36, 95% CI 0.14–0.94, *p *= 0.03); however, this risk was no longer significant in multivariate analysis (Table 3).

The combined sequential action of thrombin, factor XIIIa, and plasmin after tissue restoration results in thrombus degeneration.^7^^,^^34^ This process releases fibrin degradation product (FDP) and D-dimer, which represents the final product of the fibrinolytic system and thus plays a significant role in the clinical evaluation of thrombophilia after major surgical procedures.^34^ The dynamics of D-dimer on days 1, 2, 5, and 10 are presented in Fig. 2. Univariate analysis showed a significantly increased risk for VTE within 6 months of CRS and HIPEC for patients with elevated D-dimer on days 1 and 2, but, interestingly, days 5 and 10 were not significant. The risk in elevated day 2 D-dimer remains highly significant after performing multivariate regression (Table 3). Whether this early rise in D-dimer is related to the surgical trauma and HIPEC or if it is a marker of an underlying susceptibility to VTE development is difficult to ascertain. However, it is likely that subclinical thrombogenicity can play a role here. Nonetheless, this early rise in D-dimer is a response to the surgical treatment that might have a triggering effect on the VTE risk. A later rise in D-dimer did not predict VTE, which is probably because the later rise in D-dimer may be more related to the general postoperative inflammatory response. An assumption can be made that screening upon discharge can be abandoned since this seems to be uninformative. Loscalzo and Schafer argued that such routine screening may even be counterproductive when it leads to additional costs from surgery delays or from follow-up testing that could be avoided.^7^

Cancer-related VTE risk factors such as primary tumor classification, previous CRS, and preoperative chemotherapy did not show any relevant risks and the two main groups, appendix and colorectal, had similar VTE rates (Table 3).

One treatment-related variable was related to increased VTE, namely an incomplete cytoreduction (CC) score. The difference was between CC0–1 and CC2–3. As such, the real increase in risk occurred in patients with remaining bulky peritoneal disease. This is relevant in patients with large pseudomyxoma peritonei tumors.

In fact, some patients are treated in two stages when the Peritoneal Cancer Index (PCI)^35^ is significantly increased. When considering this alternative, assessing the postoperative VTE risk may be important. Postoperative prophylaxis may need to continue between the two-stage CRS and HIPEC treatments. The PCI score showed no significant risk for postoperative thromboembolism. Duration of surgery, perioperative blood loss, liver resection, or splenectomy showed no significant association with VTE within 6 months of CRS and HIPEC. Multivariate analysis performed by Rottenstreich et al. identified the lack of extended anticoagulation treatment at discharge as the only risk factor for thrombosis post CRS and HIPEC.^24^ However, none of the patients included in our study lacked postoperative anticoagulant treatment and none were treated for < 2 weeks. Those few patients treated with short-course postoperative anticoagulant treatment (2–3 weeks) did not develop VTE, but this is too few a number to comment about the length of treatment. Considering that only two VTEs occurred within 10 days and the remainder occurred later, it seems that a prophylaxis of 4 weeks is still recommended.

A limitation of this study is that the endpoint was assessed through the national diagnosis registry without details on positive or negative radiological examinations. The assessment of VTE most likely meant that the study may have potentially missed VTEs that have not led to hospitalization, meaning that only minor VTE events were possibly missed. Furthermore, the assessment through the national Swedish In-Patient Registry means that the study has the same follow-up for all patients. This follow-up can mainly identify symptomatic VTEs since no radiological screening was used and there is no information regarding negative radiological examinations. However, on the positive side, we do not anticipate any skewing in the follow-up process as it is similar for all referred patients regardless of where in Sweden they live. One other limitation is that the study would have been improved if the D-dimer was determined at baseline. Patients already demonstrating an increase in D-dimer at baseline may correlate with the postoperative day 2 D-dimer, highlighting possible subclinical susceptibility to VTE instead of being related to the surgical treatment. However, the D-dimer response to surgical treatment is still a relevant finding regardless of the correlation to underlying preoperative susceptibility, since the surgical treatment adds risk to this susceptibility, leading to higher incidences of clinically significant VTEs. Identifying these individuals may be important to prevent VTE development. Further studies evaluating the D-dimer response to the surgical trauma may be an important future direction in research. Considering that D-dimer has been linked to cancer prognosis and that the postoperative inflammatory environment in the abdomen may promote peritoneal recurrence, a number of important questions remain regarding this biomarker.^36^^,^^37^

## Conclusion

The incidence of symptomatic VTE within 6 months of CRS and HIPEC was 6%, which is no higher than after comparable abdominal cancer surgery. D-dimer on day 2 and incomplete cytoreduction of CC2–3 might identify patients at high risk for a VTE within 6 months of surgery who may be in need of prolonged prophylaxis and surveillance.
